# miR-1246 in tumor extracellular vesicles promotes metastasis *via* increased tumor cell adhesion and endothelial cell barrier destruction

**DOI:** 10.3389/fonc.2023.973871

**Published:** 2023-04-12

**Authors:** Masahiro Morimoto, Nako Maishi, Takuya Tsumita, Mohammad Towfik Alam, Hiroshi Kikuchi, Yasuhiro Hida, Yusuke Yoshioka, Takahiro Ochiya, Dorcas A. Annan, Ryo Takeda, Yoshimasa Kitagawa, Kyoko Hida

**Affiliations:** ^1^ Department of Vascular Biology and Molecular Pathology, Hokkaido University Graduate School of Dental Medicine, Sapporo, Japan; ^2^ Department of Oral Diagnosis and Medicine, Hokkaido University Graduate School of Dental Medicine, Sapporo, Japan; ^3^ Department of Renal and Genitourinary Surgery, Hokkaido University Graduate School of Medicine, Sapporo, Japan; ^4^ Department of Cardiovascular and Thoracic Surgery, Hokkaido University Faculty of Medicine, Sapporo, Japan; ^5^ Institute of Medical Science, Tokyo Medical University, Tokyo, Japan

**Keywords:** miRNA, extracellular vesicles, metastasis, tumor endothelial cell, VE-cadherin, ICAM-1

## Abstract

**Background:**

Tumor blood vessels play a key role in tumor metastasis. We have previously reported that tumor endothelial cells (TECs) exhibit abnormalities compared to normal endothelial cells. However, it is unclear how TECs acquire these abnormalities. Tumor cells secrete extracellular vesicles (EVs) to create a suitable environment for themselves. We have previously identified miR-1246 to be more abundant in high metastatic melanoma EVs than in low metastatic melanoma EVs. In the current study, we focused on miR-1246 as primarily responsible for acquiring abnormalities in TECs and examined whether the alteration of endothelial cell (EC) character by miR-1246 promotes cancer metastasis.

**Methods:**

We analyzed the effect of miR-1246 in metastatic melanoma, A375SM-EVs, *in vivo* metastasis. The role of tumor EV-miR-1246 in the adhesion between ECs and tumor cells and the EC barrier was addressed. Changes in the expression of adhesion molecule and endothelial permeability were examined.

**Results:**

Intravenous administration of A375SM-EVs induced tumor cell colonization in the lung resulting in lung metastasis. In contrast, miR-1246 knockdown in A375SM decreased lung metastasis *in vivo*. miR-1246 transfection in ECs increased the expression of adhesion molecule ICAM-1 *via* activation of STAT3, followed by increased tumor cell adhesion to ECs. Furthermore, the expression of VE-Cadherin was downregulated in miR-1246 overexpressed EC. A375SM-EV treatment enhanced endothelial permeability. VE-Cadherin was validated as the potential target gene of miR-1246 *via* the target gene prediction database and 3′ UTR assay.

**Conclusion:**

miR-1246 in high metastatic tumor EVs promotes lung metastasis by inducing the adhesion of tumor cells to ECs and destroying the EC barrier.

## 1 Introduction

The supply of nutrients and oxygen from blood vessels is crucial for tumor growth; thus, angiogenesis (formation of new blood vessels) is a key process involved in tumor progression (1). Furthermore, tumor blood vessels play a vital role in tumor metastasis (2, 3). Tumor blood vessels behave physiologically and morphologically different from normal blood vessels, such as lower pericyte coverage or hyperpermeability (4). We have previously reported that tumor endothelial cells (TECs), which constitute the inside of tumor vessels, have various abnormalities such as chromosomal abnormality (5, 6) and drug resistance (7) compared to normal endothelial cells (ECs). However, it is unclear how TECs acquire these abnormalities. We have reported that TEC isolated from high metastatic tumors showed more proangiogenic, drug-resistant, and stem-like phenotype than TEC isolated from low metastatic ones (8). Interestingly high metastatic tumor cell-conditioned media induced these phenotypes in endothelial cells (7). Thus, we assumed that the tumor-secreting factors might induce the TEC’s abnormalities.

Extracellular vesicles (EVs) are covered with the lipid bilayer and are secreted by various cells. They contain biomolecules such as protein, mRNA, and miRNA (9). We have reported that tumor cell-derived EVs were incorporated into ECs, enhancing angiogenesis *via* Akt activation (10). EVs play a significant role in intracellular communication, and it is reported that tumor cell-secreting EVs affect tumor growth and metastasis (11).

miRNA, a non-coding RNA having approximately 20 nucleotides, binds to the target mRNA, destabilizes it, and inhibits protein production *via* suppressing translation (12). They are transported between cells as contents of EVs and alter the properties of recipient cells. For example, miR-210 in tumor EVs has been shown to induce angiogenesis (13), and miR-105 in tumor EVs promotes metastasis by inhibiting ZO-1, which is an adhesion molecule between ECs (14). We hypothesized that miRNA in EVs secreted by tumor cells acts as a tumor microenvironment responsible for the acquisition of TEC abnormality. We identified that miR-1246 is more abundant in high metastatic tumor EVs than in low metastatic ones, and that miR-1246 elucidated the mechanism of drug resistance acquisition by IL-6 secretion of ECs (15). However, there are only limited reports on miR-1246 and metastasis.

During metastasis, tumor cells adhere to ECs by migration and invasion from the primary site and penetrate the blood vessels through ECs. Subsequently, tumor cells circulate in the body *via* the bloodstream, adhere to ECs of distant organs, and form metastatic lesions *via* extravasation (16). In the present study, we addressed the role of tumor EV-miR-1246 in the adhesion between ECs and tumor cells and the EC barrier to determine the involvement of EV-miR-1246 transfer to ECs in tumor metastasis.

## 2 Materials and methods

### 2.1 Cell lines and culture conditions

Human dermis microvascular endothelial cells (HMVECs) and human umbilical vein endothelial cells (HUVECs) were obtained from Lonza (Basel, Switzerland). Immortalized HMVECs (iHMVECs) and immortalized HUVECs (iHUVECs) were established by transducting SV40 large T antigen and hTERT as previously described (17). Briefly, lentiviruses were produced using HEK293T cells co-transfected with packaging constructs pCAG-HIVgp and the VSV-G- and REV-expressing construct pCMV-VSV-G-RSV-REV, pCSII-CMV-hTERT-IRES2-Venus, or pRRLsin-SV40 T antigen-IRES-mCherry. Each EC was analyzed and sorted by flow cytometry (FACSAria II) with Venus and mCherry signals to purify transducted cells. iHMVECs were cultured in the EC growth medium for microvascular cells (EGM-2MV) (Lonza), whereas HUVECs and iHUVECs were cultured in the EC growth medium (EGM-2) (Lonza). A375 cells were obtained from American Type Culture Collection (Manassas, VA, USA). A375SM cells (super-metastatic human melanoma cells) were kindly supplied by Dr. Fidler (M.D. Anderson Cancer Center, Houston, TX, USA) (18). The miRZip-1246 Anti-miR-1246 microRNA construct and Scramble Hairpin Control Anti-MicroRNA Construct (System Biosciences, CA, USA) were used to establish the Anti-miR-1246 A375SM and Control-miR A375SM cell lines, respectively. For the establishment of tdTomato-luc2 A375 and tdTomato-luc2 A375SM cell, ptdTomato-C1 vector (Clontech, Clontech, Palo Alto, CA, USA) and pGL4.50 [luc2/CMV/Hygro] vector (Promega, Madison, WI, USA) were inserted into the EcoRI-Notl region of pCSII-CMV-MCS to create pCSII-CMV-tdTomato-luc2 vector. Each lentiviral vector was transfected into HEK293T by FuGENE HD Transfection Reagent, along with the packaging vector pCAG-HIVgp and the VSV-G- and REV-expressing construct pCMV-VSV-G-RSV-REV (from H. Miyoshi, Department of Physiology, Keio University School of Medicine, Tokyo, Japan) according to the manufacturer’s instructions. A375 and A375SM cells transduced by each construct described above were cultured in minimum essential medium (GIBCO, Grand Island, NY, USA) supplemented with 10% heat-inactivated fetal bovine serum (FBS). All cells were cultured at 37°C in a humidified atmosphere containing 5% CO_2_ and 95% air.

### 2.2 Mice

Six-week-old female nude mice (BALB/c AJcl-nu/nu; Clea, Japan) were housed under specific pathogen-free conditions. All animal care and experimentation procedures adhered to institutional guidelines and were approved by the Ethical Committee for Experimental Animal Care of Hokkaido University.

### 2.3 Preparation of EVs

Tumor cells (1 × 10^6^ cells) were cultured for 48 h in each medium with 10% EV-depleted FBS. EV-depleted FBS was prepared by ultracentrifugation at 100,000×*g* for 16 h at 4°C (Optima XPN-80 and SW32Ti; Beckman Coulter, Miami, FL, USA). The medium was collected and centrifuged at 2,000×*g* for 10 min at 4°C, and the supernatant was filtered with a 0.2-µm filter unit (Nalgene; Thermo Scientific, MA, USA) to remove the debris. The filtered medium was ultracentrifuged at 175,000×*g* for 84 min at 4°C (Optima XPN-80 and SW32Ti) to isolate the EVs. The pellet was washed with PBS and ultracentrifuged under the same condition, and EVs were eluted in PBS. For EV preparation from serum, the ultracentrifugation was conducted at 210,000×*g* for 40 min at 4°C (Optima XPN-80 and SW55Ti). The micro BCA protein assay kit (Thermo Scientific) was used to estimate the protein concentration. For EVs treatment, 3 µg of EVs was used per 1.0 × 10^5^ cells unless otherwise stated. The same amount of PBS was used as the control.

### 2.4 Characterization of EVs

The observation of A375SM-EVs by transmission electron microscope (JEM-1400; JEOL, Tokyo, Japan) was performed as previously described (15) (**Figure S1A**). The size of EVs was measured using Dynamic Light Scattering with a Zetasizer Nano ZS (Malvern Instruments, Southborough, UK) (**Figure S1B**). Western blotting was used with anti-HSP70 (1:1,000, BD Pharmingen, USA, #556433) and anti-Cytochrome C (1:500, BD Bioscience, #610607) antibodies. A375SM-EVs expressed the EV marker HSP70, but not cytochrome C, a cytoplasm protein (**Figure S1C**). Our experimental data have been submitted to EV-TRACK knowledgebase (EV-TRACK ID: 220296).

### 2.5 RNA isolation and real-time quantitative reverse-transcription PCR

Total RNA was isolated with ReliaPrep RNA Cell Miniprep System (Promega). As previously described, first-strand cDNA was synthesized using ReverTra-Plus (Toyobo, Osaka, Japan) (19). Real-time qRT-PCR was performed using the KAPA SYBR FAST qPCR Kit (KAPA Biosystems, Boston, MA, USA) according to the manufacturer’s instructions. Cycling conditions were set based on CFX Manager (Bio-Rad, Hercules, CA, USA). Relative mRNA expression levels were normalized to glyceraldehyde 3-phosphate dehydrogenase (GAPDH) and analyzed using the delta-delta-Ct method. The primer sequences were as follows:

human GAPDH: forward, 5′-ACAGTCAGCCGCATCTTCTT-3′; reverse, 5′-GCCCAATACGACCAAATCC-3′, human ICAM-1: forward, 5′-GGCAAGAACCTTACCCTACGCTGCC-3′; reverse, 5′-GTTCAGTGCGGCACGAGAAATTGGC-3′, human VE-Cadherin: forward, 5′-AAGTACAGCATCTTGCGGGGCGAC-3′; reverse, 5′-TTGATGATGCCCTCGTTGTGGGCG-3′, human Claudin-5: forward, 5′-AAGATTGAGAGCTGCCAGAGGC-3′; reverse, 5′-TACCCTCTTTGAAGGTTCGGGG-3′, human ZO-1: forward, 5′-GGGGAGGGTGAAGTGAAGA-3′; reverse, 5′-AGGCATTTCTGCTGGTTAGTATG-3.

### 2.6 Analysis of miRNA level

Total RNA was isolated from the cells or EVs using TRIzol reagent (Invitrogen, Carlsbad, CA, USA) and RNeasy Mini Kit (Qiagen, Valencia, CA, USA). Cel-miR-39 (Hokkaido system science, Japan) was added (final concentration: 1 nM) to EVs solution. Reverse transcription was performed using the Taqman miRNA reverse transcription kit (Applied Biosystems, Carlsbad, CA, USA). miRNA expression levels were examined by qRT-PCR using Universal PCR Master Mix II (Applied Biosystems) and TaqMan MicroRNA Assays (Applied Biosystems) according to the manufacturer’s instructions. Cycling conditions were set based on CFX Manager (Bio-Rad). Relative miRNA expression levels were normalized to cel-miR-39 as external control. Primers were defined as follows:

miR-1246 (Assay ID: CSN1EFS), cel-miR-39 (Assay ID: 000200; UCACCGGGUGUAAAUCAGCUUG).

### 2.7 miRNA mimic transfection

ECs were seeded at 1 × 10^5^ cells/well (6-well plate), followed by transfection with 50 nM miRIDIAN microRNA Human hsa-miR-1246-Mimic (CN-001040, Dharmacon; GE Dharmacon, Lafayette, CO, USA) using Lipofectamine RNAiMAX (Invitrogen) to a final concentration of 25 nM. miRIDIAN microRNA Mimic Negative Control (CN-001000-01-05; Dharmacon) was used as a negative control. After 6 h, the medium was changed and used for subsequent analysis.

### 2.8 Immunocytochemistry

miR-1246 mimic transfected iHMVECs were fixed with 4% paraformaldehyde (PFA) at room temperature for 10 min. After blocking with 5% goat serum in PBS for 1 h, the cells were incubated with mouse anti-human CD144 antibody (1:400, 555661; BD Pharmingen) at 4°C overnight. After washing with PBS, cells were incubated with Alexa Flour 594 goat anti-mouse antibody (1:400, 11032; Invitrogen) at room temperature for 2 h. Counterstaining was performed using DAPI (Dojin, Kumamoto, Japan) and mounted with Perma Fluor (Thermo Scientific). The stained sample was observed using a confocal microscope (FV10i; Olympus). Fluoview FV10-ASM Viewer Software (Olympus) was used for image processing. The stained area was calculated using ImageJ (National Institutes of Health, Bethesda, MD, USA) from 8 randomly selected fields of view.

### 2.9 Tumor cell adhesion assay

iHMVECs with miR-1246 mimic transfection or EV treatment (10 µg) were incubated for 48 h on a 35-mm dish to form a monolayer. tdTomato-luc2 A375 or A375SM cells (2.0 × 10^5^ cells) were added to iHMVEC monolayers and incubated for 3 h. After removing non-adherent tumor cells by washing with PBS three times, cells were fixed with 4% PFA. Adherent tumor cells were observed with a confocal microscope (FV10i). Fluoview FV10-ASM Viewer Software was used for image processing. The number of adherent tumor cells was counted from 5 randomly selected fields using ImageJ.

### 2.10 *In vitro* permeability assay

iHUVECs were seeded at 5.0 × 10^4^ cells on 0.4 µm pore size Transwell filters (Corning, 3413, NY, USA) and incubated for 72 h to allow monolayer formation. After EVs treatment (3 µg), FITC-dextran (Sigma-Aldrich, FD70S) was added to the top well at 1 mg/ml and incubated for 30 min at room temperature. Then, 100 µl of the medium in the bottom well was transferred into a Nunc^®^ MicroWell 96-Well Optical-Bottom Plates with Polymer Base (Thermo Scientific, 165305). The fluorescence intensity was measured with a Varioskan Flash (Thermo Scientific).

### 2.11 Western blotting

The cells were lysed and collected in radioimmunoprecipitation assay buffer (Cell Signaling Technology, Beverly, MA, USA). The total protein concentration was determined using the BCA Protein Assay Kit (Pierce, Rockford, IL, USA). An equal amount of proteins were separated by sodium dodecyl sulfate-polyacrylamide gel electrophoresis and transferred to polyvinylidene difluoride membranes. The membrane was blocked with 5% skim milk/TBST for 60 min. The antibodies used are specific for STAT3 (1:1,000, Cell Signaling Technology, 12640S, Beverly, MA, USA), pSTAT3 (1:1,000, Cell Signaling Technology, 9145S), VE-Cadherin (1:1,000, Abcam, ab33168, Cambridge, UK), ICAM-1 (1:2,000, Abcam, ab53013), and β-actin (1:5,000, Cell Signaling Technology, 4970), with anti-rabbit HRP-labeled antibody as the secondary antibody (1:5,000, Cell Signaling Technology, 7074). The signals were developed using ECL Western Blotting Detection Reagent (GE Healthcare, Little Chalfont, UK) and detected using LAS-4000 mini image analyzer (FUJIFILM, Tokyo, Japan). The levels of pSTAT3, VE-Cadherin and ICAM-1 were normalized to that of β-actin by densitometry using Image J.

### 2.12 3′ UTR assay

Each vector plasmid combined with miRNA mimic transfection was performed with DharmaFECT Duo reagent (Horizon Discovery), in accordance with the manufacturer’s protocol and then, HEK293 cells were collected 48 h after co-transfection with the 3’UTR assay vector (pmiR-CDH5) and miR-1246 mimic (Dharmacon), and luciferase activity was measured using the the Dual-Glo Luciferase Assay System (Promega) in accordance with the manufacturer’s protocol. Firefly luciferase activity was normalized to Renilla luciferase activity.

A 822 bp fragment from the 3’UTR of TET2 containing the predicted target sequence of miR-1246 (located at positions 924-930 of the TET2 3’UTR) were PCR-cloned from 3’ UTR clone of CDH5 for miRNA target validation (Origene, Rockville, MD, USA). Three prime A-overhang was added to the PCR products after 15 minutes of regular Taq polymerase treatment at 72˚C. The PCR products were cloned into a pGEM-T easy vector (Promega). The amplified products were ligated into the NheI and SalI sites of the 3’UTR of the firefly luciferase gene in the pmirGLO Dual-Luciferase miRNA Target Expression vector (Promega) to generate pmiR-CDH5. Primer sequences are as follows (shown 5’ to 3’): CDH5_F, CAGCTAGCTTCTCTGGAGAAGGCCTGGAAG and CDH5_R, CAGTCGACTATTGCCCAGGCTAAAGATTTT. Site-directed mutagenesis was performed in the seed sequences of CDH5. PrimeStar Max DNA Polymerase (Takara, Japan) was used for PCR amplification. Forward primer and reverse primer sequences are as follows (shown 5’ to 3’): CDH_Mut_F, CGCCTAACGAAGCTCTCTTTCTTTTCTCT and CDH5_Mut_R, GAGCTTCGTTAGGCGACCAGGTGAGGCAG.

### 2.13 Transendothelial electrical resistance assay

Millicell hanging cell culture inserts with a pore size of 0.4 µm (MCHT24H48; Millipore, Bedford, MA, USA) were set on a 24-well plate, and HUVECs were seeded at 4 × 10^4^ cells on the insert. The medium-only wells were prepared for correction. The medium was changed after 48 h. A375SM-EVs treatment (3 µg) or miR-1246 transfection was performed 72 h after seeding, followed by TEER measurement. TEER was measured 24 h after its treatment. Millicell ERS-2 (Millipore) was used to measure TEER. The formula of TEER value was calculated as: (resistance of experimental wells − resistance of empty wells) Ω × 0.33 cm^2^ (the membrane area of the cell culture insert).

### 2.14 STAT3 inhibitor treatment

2 × 10^5^ iHMVECs were seeded in 6-well plates, followed by 24 h starvation with 5%FBS-EBM2. ECs were pretreated with STAT3 phosphorylation inhibitor (S3I-201, Calbiochem) for 2 h. Then, ECs were treated with A375SM-EV (2μg) and S3I-201 (10 or 50 μM, vehicle: DMSO). These concentrations were based on previous reports (20, 21). Total RNA was extracted 12 h later for further mRNA expression analysis.

### 2.15 *In vivo* tumor metastasis model

In the experimental group (EV injection), A375SM-EV (3 µg) was injected intravenously to mice twice a week (five times in total), and then tdTomato-luc2 A375 (2.0 × 10^5^ cells) was injected intravenously. In the control group, PBS was used instead of EVs. After 24 h, VivoGlo Luciferin, *In Vivo* Grade (Promega), was intraperitoneally administered, and the tumor cell signals were detected using IVIS Spectrum (Caliper Life Sciences, Hopkinton, MA, USA) to analyze tumor cell adhesion to the lung. Forty days after injection, tumor cell signals were measured to analyze lung metastasis. The excised lungs were stained with H-E staining, and the number of metastatic lesions was counted.

Anti-miR-1246 A375SM cells (1.0 × 10^6^) or Control-miR A375SM cells (1.0 × 10^6^) were suspended in HBSS and subcutaneously injected into the right flanks of mice to analyze the effect of miR-1246 in tumor metastasis. The serum was used to analyze the EV-miR-1246 level after 28 days, the lungs were removed, and the GFP signals expressed in tumor cells were evaluated using IVIS Spectrum.

### 2.16 *In vivo* permeability assay

Anti-miR-1246 A375SM cells (1.0 × 10^6^) or Control-miR A375SM cells (1.0 × 10^6^) were suspended in HBSS and subcutaneously injected into the right flank of mice. After 4 weeks, Rhodamine-dextran (MW 70,000, Invitrogen, D1841) was administered intravenously 3 h before transcardiac perfusion. The tumors removed were sectioned after making a frozen block using the Tissue-Tek OCT compound (Sakura Finetek, Torrance, CA, USA). The frozen sections were stained with DAPI for 10 min and then observed using a BZ-X810 microscope (Keyence Corporation, Itasca, IL, USA). Rhodamine signal was quantified using BZ-X800 Analyzer software (Keyence).

### 2.17 Statistical analysis

Statistical analysis was performed using JMP version 13 (SAS Institute). All the data are presented as mean ± standard deviation unless otherwise stated. The Student’s t-test or Wilcoxon test was used for comparing the two groups. Differences among groups were determined using one-way ANOVA, followed by a Tukey–Kramer multiple comparison test. A P-value <0.05 was considered to be significant.

## 3 Results

### 3.1 High metastatic tumor EVs induce tumor cell adhesion to the lung and promote lung metastasis

We analyzed the effects of tumor EVs on ECs in lung metastasis using a high metastatic tumor cell line, A375SM. We have previously reported the differential effects of tumor EVs on ECs, and the ECs’ phenotype is altered to a drug-resistant or proangiogenic phenotype (8, 15). It has been reported that tumor EVs induced metastasis (14, 22).

**Figure 1:**
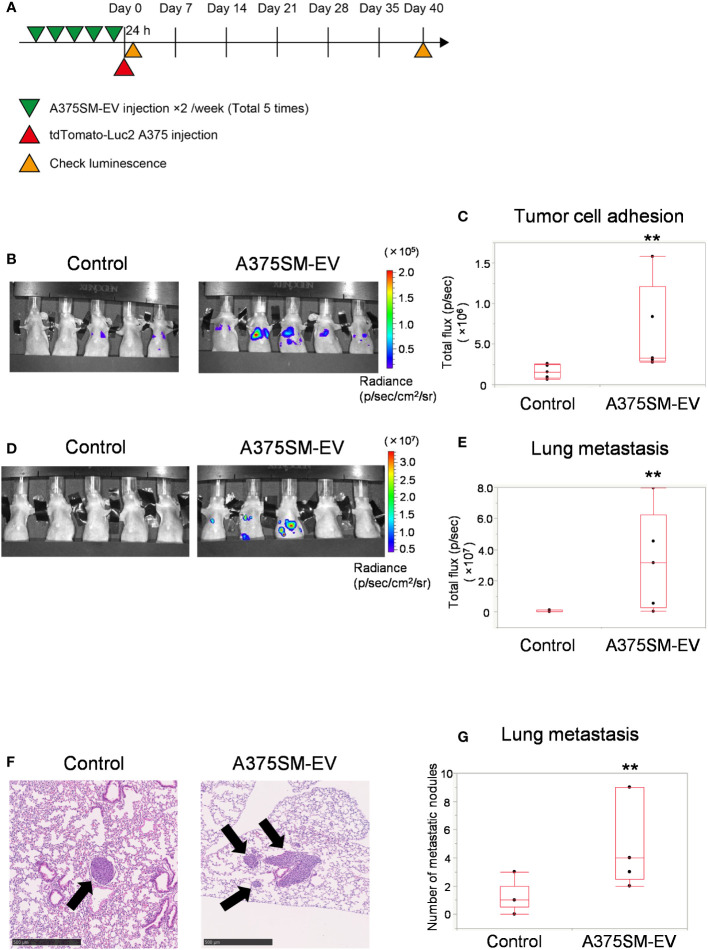
High metastatic tumor EVs induce tumor cell adhesion to the lung and promote lung metastasis. **(A)** The experimental design of tumor EV and tumor cell injection. Tumor cells were injected after five injections of A375SM-EVs. After 24 h and 40 days, tumor cell signals were analyzed using an *in vivo* imaging system (IVIS). **(B, C)** Tumor cell luminescence intensity in the lungs of the control and A375SM-EV injected groups was detected using IVIS Spectrum 24 h after tumor injection **(B)**. Quantitative analysis of luminescence intensity (Total flux) was shown in **(C)** (**P = 0.012, Wilcoxon test; n = 5 mice per group). **(D)** Tumor cell luminescence intensity in the control and A375SM-EV injected groups after 40 days from tumor injection was detected using IVIS Spectrum. Quantitative analysis of luminescence intensity (Total flux) was shown in **(E)** (**P = 0.037, Wilcoxon test; n = 5 mice per group). **(F)** Representative H-E staining images of lung metastases in the two groups. Arrows indicate tumor nodules. **(G)** The number of metastatic nodules in the whole lung tissue was quantified (**P = 0.026, Wilcoxon test; n = 5 mice per group).

The tumor cell adhesion to the lung and lung metastasis was analyzed to address the alteration of the metastatic potential of a low metastatic tumor cell line, A375, after pretreating EVs isolated from A375SM (**Figure 1A**). The A375SM-EV treated group had more signals of tumor cells in the lung than the control group 24 h after tumor cell injection (**Figures 1B, C**), suggesting that tumor adhesion to the lung was stimulated by pretreatment of A375SM-EV. Similarly, A375SM-EV treated mice showed increased lung metastasis 40 days after tumor cell injection (**Figures 1D, E**). The A375SM-EV group showed more metastasis nodules in the lung than the control group in the histological study (**Figures 1F, G**). The above results suggest that EVs from high metastatic tumors induce tumor cell adhesion to the lung and promote lung metastasis.

### 3.2 miR-1246 knockdown of tumor cells reduces lung metastasis

**Figure 2:**
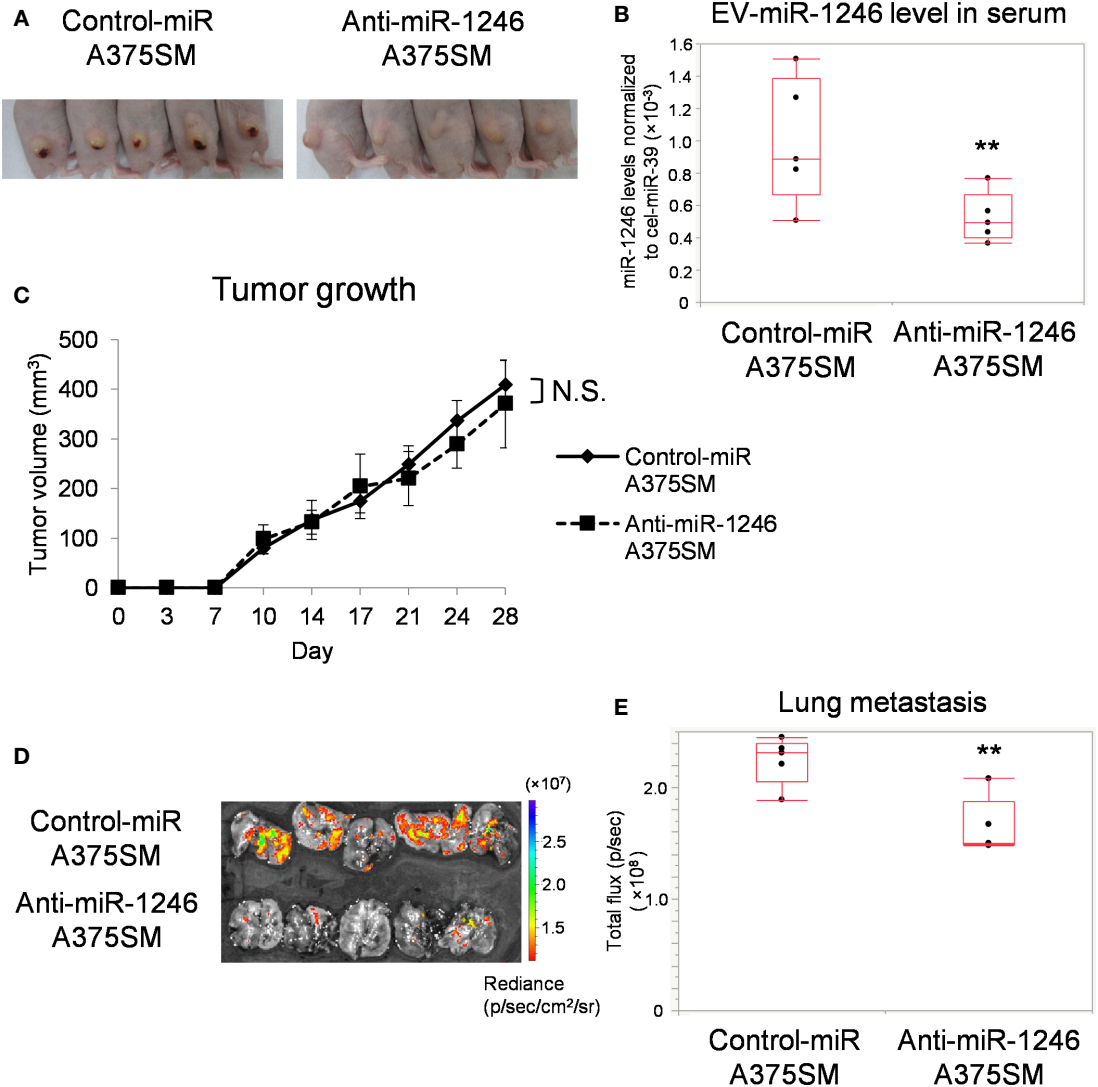
miR-1246 knockdown of tumor cells reduces lung metastasis. **(A)** Images of mice, 28 days after subcutaneous injection of Control-miR A375SM and Anti-miR-1246 A375SM. **(B)** miR-1246 levels in serum EVs from Control-miR A375SM or Anti-miR-1246 A375SM tumor-bearing mice. Sera were collected 28 days after subcutaneous injection of each tumor (**P = 0.037, Wilcoxon test; data are presented as mean ± SD; n = 3 real-time RT-PCR runs, n = 5 mice per group). **(C)** Tumor volume was assessed using the formula: (width^2^ × length)/2 (mm^3^). Two-sided Student’s t-test. N.S.: not significant. P = 0.42 **(D)** Tumor cell fluorescence intensity (GFP signal) in the lungs was detected using IVIS Spectrum. Quantitative analysis of fluorescence intensity (Total flux) was shown in **(E)** (**P = 0.022, Wilcoxon test; n = 5 mice per group).

We have previously reported that miR-1246 levels are higher in A375SM-derived EVs than in A375 (15). To evaluate the role of miR-1246, we established a stable miR-1246-knockdown A375SM (Anti-miR-1246 A375SM) using a lentivirus vector. miR-1246 levels in anti-miR-1246 A375SM were downregulated in cells and EVs compared to control-miR A375SM (**Figure S2**). Anti-miR-1246 A375SM and Control-miR A375SM were injected into mice subcutaneously, and their tumors and lungs were removed after 28 days (**Figure 2A**). EVs were isolated from the serum of each tumor-bearing mice, and the miR-1246 levels were significantly lower in anti-miR-1246 A375SM tumor-bearing mice than in control-miR A375SM bearing mice (**Figure 2B**). There was no significant difference in tumor growth between the two groups (**Figure 2C**). However, the GFP signal showed that the lung metastasis was significantly suppressed in Anti-miR-1246 A375SM tumor-bearing mice compared to the control-miR A375SM (**Figures 2D, E**). These findings suggest that miR-1246, which is found in EVs of the high metastatic tumor, A375SM, promotes metastasis but not tumor growth.

### 3.3 miR-1246 in high metastatic tumor EVs induces ICAM-1 in ECs *via* activation of STAT3

We studied the adhesion of tumor cells to ECs as one of the mechanisms for metastasis by miR-1246. ICAM-1 expressed on ECs is the key molecule for tumor cells to adhere to ECs (23) and is induced *via* STAT3 activation (24).

**Figure 3:**
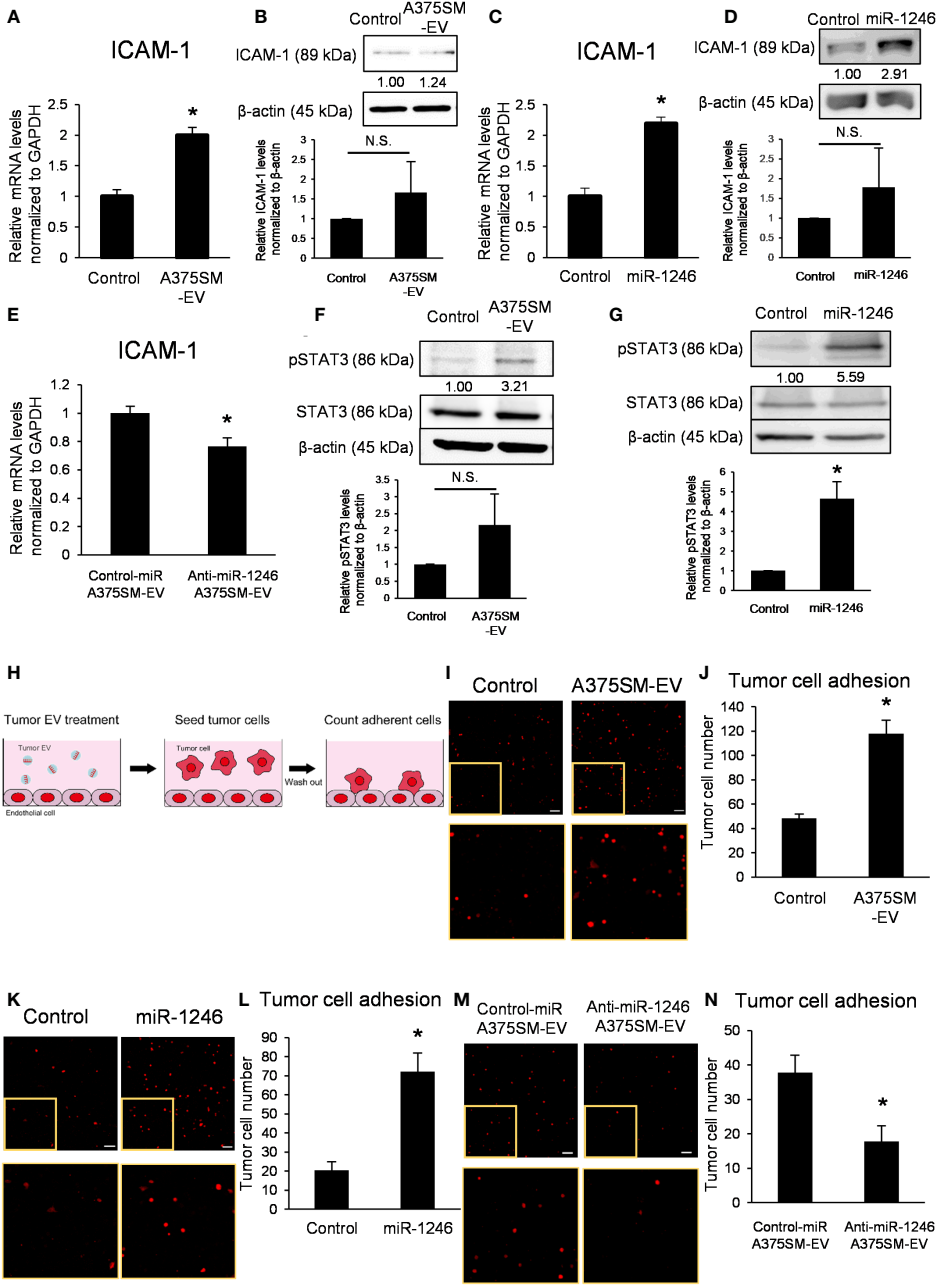
miR-1246 in high metastatic tumor EVs activates STAT3 in ECs, resulting in the induction of ICAM-1 expression. **(A)** ICAM-1 mRNA levels in iHMVECs treated with A375SM-EVs for 12 h were examined by qRT-PCR. PBS was used as the control. Data are presented as mean ± SD; n = 4 real-time RT-PCR runs (*P = 0.00012 vs. control, two-sided Student’s t-test). **(B)** The levels of ICAM-1 in iHMVECs treated with A375SM-EVs for 48 h were determined using western blotting. PBS was used as the control. β-Actin was used as an internal control. The level of ICAM-1 was normalized to that of β-actin by scanning densitometry using Image J. Data are presented as mean ± SD; n = 3 (vs. control, two-sided Student’s t-test) N.S.: not significant. P = 0.22 **(C)** ICAM-1 mRNA levels in iHMVECs 48 h after miR-1246 transfection were examined by qRT-PCR. microRNA Mimic Negative Control was used as the control. Data are presented as mean ± SD; n = 4 real-time RT-PCR runs (*P < 0.0001 vs. control, two-sided Student’s t-test). **(D)** The levels of ICAM-1 in iHMVECs 48 h after miR-1246 transfection were determined using western blotting. microRNA Mimic Negative Control was used as control. β-Actin was used as an internal control. The level of ICAM-1 was normalized to that of β-actin by scanning densitometry using Image J. Data are presented as mean ± SD; n = 3 (vs. control, two-sided Student’s t-test) N.S.: not significant. P = 0.24 **(E)** ICAM-1 mRNA levels in iHMVECs treated with Anti-miR-1246 A375SM-EVs for 12 h were examined by qRT-PCR. Data are presented as mean ± SD; n = 4 real-time RT-PCR runs (*P = 0.0019 vs. control-miR A375SM-EVs, two-sided Student’s t-test). **(F)** The levels of pSTAT3 in iHMVECs treated with A375SM-EVs for 30 min were determined by western blotting. STAT3 and β-actin were used as an internal control. The level of pSTAT3 was normalized to that of β-actin by scanning densitometry using Image J. Data are presented as mean ± SD; n = 3 (vs. control, two-sided Student’s t-test) N.S.: not significant. P = 0.094 **(G)** The levels of pSTAT3 in iHMVECs 24 h after miR-1246 transfection were determined by western blotting. microRNA Mimic Negative Control was used as the control. STAT3 and β-actin were used as an internal control. The level of pSTAT3 was normalized to that of β-actin by scanning densitometry using Image J. Data are presented as mean ± SD; n = 3 (*P = 0.0018 vs. control, two-sided Student’s t-test) **(H)** The schematic of adhesion assay. **(I)** Representative fluorescent images of adherent tdTomato-luc2-expressing A375 cells to iHMVEC monolayer treated with A375SM-EVs. The lower panel shows an enlarged image of the regions marked with yellow rectangles. PBS was used as the control. **(J)** The number of tdTomato-luc2 A375 cells was counted. Data are presented as mean ± SD; n = 5 fields. Scale bar: 100 µm (*P < 0.0001 vs. control, two-sided Student’s t-test). **(K)** Representative fluorescent images of adherent tdTomato-luc2-expressing A375 cells to miR-1246 transfected iHMVEC monolayer. The lower panel shows an enlarged image of the regions marked with yellow rectangles. microRNA Mimic Negative Control was used as the control. **(L)** The number of tdTomato-luc2 A375 cells was counted. Data are presented as mean ± SD; n = 5 fields. Scale bar: 100 µm (*P < 0.0001 vs. control, two-sided Student’s t-test). **(M)** Representative fluorescent images of adherent tdTomato-luc2-expressing A375 cells to iHMVEC monolayer treated with Anti-miR-1246 A375SM-EVs. The lower panel shows an enlarged image of the regions marked with yellow rectangles. **(N)** The number of tdTomato-luc2 A375 cells was counted. Data are presented as mean ± SD; n = 5 fields. Scale bar: 100 µm (*P = 0.00018 vs. control-miR A375SM-EVs, two-sided Student’s t-test).

A375SM-EV treatment upregulated mRNA and protein expressions of ICAM-1 in ECs (**Figures 3A, B**); similarly, miR-1246 increased ICAM-1 expression level (**Figures 3C, D**). On the contrary, anti-miR-1246 A375SM-EVs decreased the expression level of ICAM-1 in ECs (**Figure 3E**).

Furthermore, A375SM-EV treatment and miR-1246 transfection activated STAT3 in ECs (**Figures 3F, G**). STAT3 inhibitor cancelled the induction of ICAM-1 by A375SM-EV treatment (**Figure S3**). These results demonstrated that miR-1246 in high metastatic tumor EVs increased ICAM-1 expression *via* the activation of STAT3. An adhesion assay was performed to investigate the effect of miR-1246 in EVs on tumor cells (**Figure 3H**). Both A375SM-EV treatment and miR-1246 transfection promoted A375 adhesion to ECs (**Figures 3I–L**). On the other hand, the number of A375 cells that adhered to ECs decreased upon anti-miR-1246 A375SM-EVs treatment (**Figures 3M, N**). In addition, even when high metastatic A375SM cells were seeded on the EC monolayer, both A375SM-EV treatment and miR-1246 transfection promoted their adhesion to ECs (**Figures S4A–D**). On the contrary, anti-miR-1246 A375SM-EVs treatment reduced A375SM adhesion to ECs (**Figures S4E, F**). These findings suggest that miR-1246 in high metastatic tumor EVs increases adhesion between tumor cells and ECs *via* ICAM-1 induction.

### 3.4 miR-1246 suppresses VE-Cadherin expression and destroys EC barrier functions

We next focused on vascular barrier dysfunction as a mechanism for promoting metastasis by miR-1246, and the cell–cell adhesion of ECs was analyzed. EC adhesion is composed of adherence and tight junctions (25). VE-Cadherin constitutes adherens junction, whereas tight junction consists mainly of claudin-5 and zonula occludens-1 (ZO-1) (26). Disrupting the EC barrier enhances vascular permeability and promotes metastasis.

**Figure 4:**
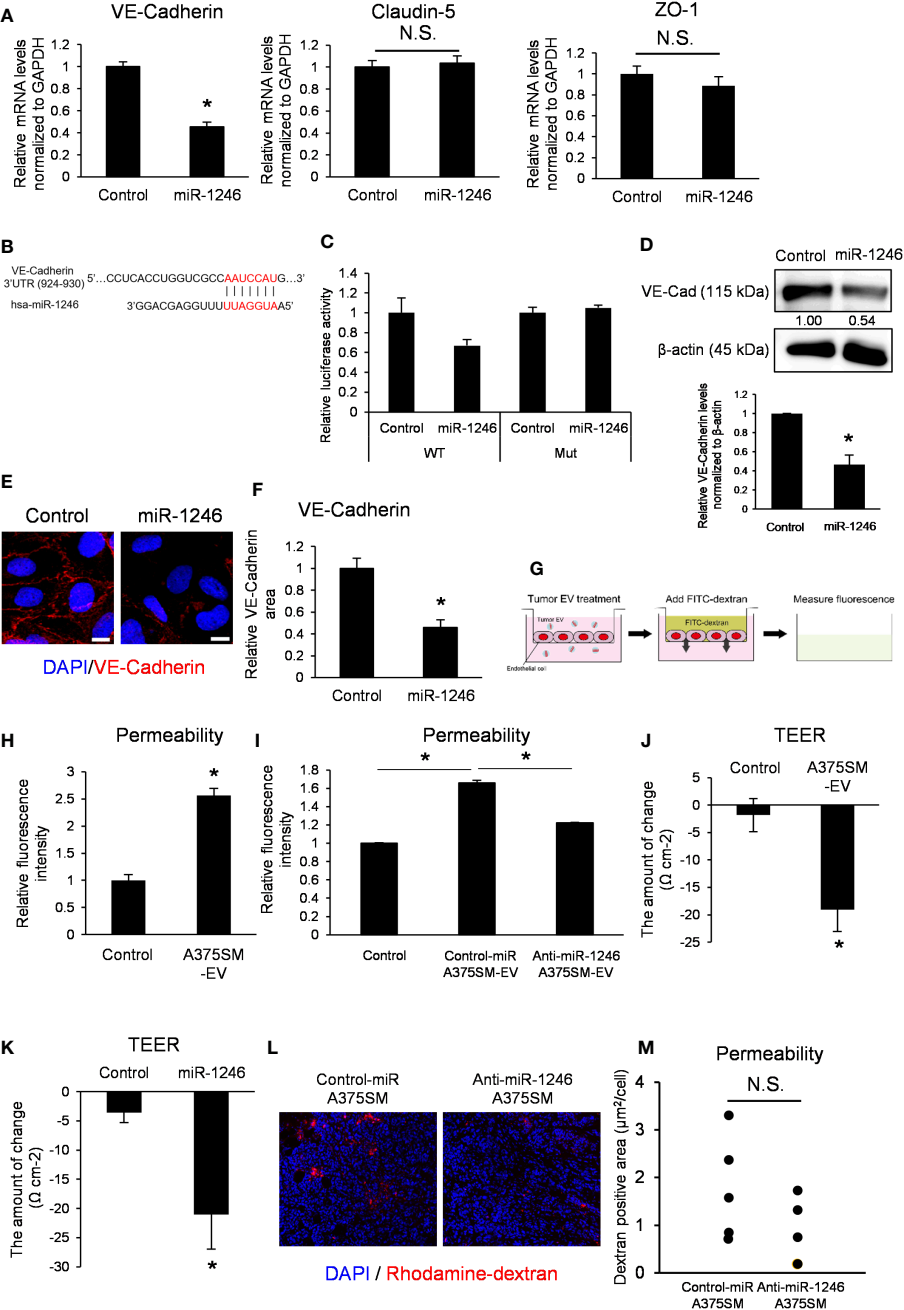
miR-1246 destroys the EC barrier through VE-Cadherin downregulation. **(A)** Expression levels of VE-Cadherin, Claudin-5, and ZO-1 mRNA in iHMVECs 48 h after miR-1246 transfection were examined by qRT-PCR. microRNA Mimic Negative Control was used as the control. Data are presented as mean ± SD; n = 4 real-time RT-PCR runs (*P = 0.0075 vs. control, two-sided Student’s t-test). N.S.: not significant. P = 0.45 (Claudin-5), 0.095 (ZO-1). **(B)** The binding position between hsa-miR-1246 and 3′ UTR of VE-Cadherin was predicted using TargetScan (http://www.targetscan.org). **(C)** Relative luciferase activities of HEK293 cells transfected with miR-1246 mimic and 3′ UTR of VE-Cadherin (P =0.088 (WT), 0.44 (Mut) vs. control, two-sided Student’s t-test). microRNA Mimic Negative Control was used as the control. Data are presented as mean ± SD; n = 4. **(D)** The levels of VE-Cadherin in miR-1246 transfected iHMVECs were determined by western blotting. microRNA Mimic Negative Control was used as the control. β-Actin was used as an internal control. The level of VE-Cadherin was normalized to that of β-actin by scanning densitometry using Image J. Data are presented as mean ± SD; n = 3 (*P = 0.00084 vs. control, two-sided Student’s t-test). **(E)** Representative images of VE-Cadherin immunostaining (red) and DAPI nuclear staining (blue) in miR-1246 transfected iHMVECs. **(F)** The quantitative analysis of the VE-Cadherin staining area in miR-1246 transfected iHMVECs **(E)** was calculated using ImageJ (*P < 0.0001 vs. control, two-sided Student’s t-test). Data are presented as mean ± SD; n = 8 fields. Scale bar: 10 µm. **(G)** The schematic of permeability assay. The leakage of FITC-dextran was measured using a microplate reader. **(H)** Permeability assay was performed in iHUVECs treated with A375SM-EVs. PBS was used as the control (*P < 0.0001 vs. control, two-sided Student’s t-test). Data are presented as mean ± SD; n = 3. **(I)** Permeability assay was performed in iHUVECs treated with Control-miR or Anti-miR-1246 A375SM-EVs (*P < 0.0001 vs. control-miR A375SM-EVs, one-way ANOVA, followed by a Tukey–Kramer multiple comparison tests). PBS was used as the control. Data are presented as mean ± SD; n = 3. **(J)** Changes of TEER in HUVECs treated with A375SM-EVs were examined by TEER assay (*P = 0.0038 vs. control, two-sided Student’s t-test). PBS was used as the control. Data are presented as mean ± SD; n = 3. **(K)** Changes of TEER in miR-1246 transfected HUVECs were examined (*P = 0.0079 vs. control, two-sided Student’s t-test). Data are presented as mean ± SD; n = 3. **(L)** Representative images of rhodamine-dextran (red) leaked in the tumors of rhodamine-dextran-injected tumor-bearing mice. Blue signals represent cell nuclei (DAPI). Quantitative analysis of leaked dextran was shown in **(M)** (two-sided Student’s t-test; n =5 fields in each tumor; n = 5 mice per group N.S.: not significant P = 0.21).

miR-1246 transfection into ECs suppressed VE-Cadherin mRNA expression but not claudin-5 or ZO-1 (**Figure 4A**). We predicted the miR-1246 target using the publicly available database for miRNA target prediction (TargetScanHuman) and found that miR-1246 may bind to the 3′ UTR of VE-Cadherin (**Figure 4B**). The 3′ UTR reporter assay showed that luciferase activity tended to be decreased in VE-cadherin 3’ UTR construct, but not in the mutant. It suggested that VE-Cadherin could be either a direct target of miR-1246 or indirectly negatively regulated (**Figure 4C**). Moreover, western blotting revealed that miR-1246 transfection reduced VE-Cadherin protein expression in ECs (**Figure 4D**). Immunocytochemistry analysis of VE-Cadherin showed that miR-1246 attenuated the VE-Cadherin signals in ECs (**Figures 4E, F**). Next, we analyzed the change in endothelial permeability by adding A375SM-EVs to the ECs monolayer (**Figure 4G**). A375SM-EV treatment increased the dextran leakage to the lower chamber, indicating enhanced endothelial permeability (**Figure 4H**), and anti-miR-1246 A375SM-EVs attenuated the leakage induced by Control-miR A375SM-EVs (**Figure 4I**). Consistent with the above results, both A375SM-EV treatment and miR-1246 transfection reduced TEER, suggesting that miR-1246 containing tumor EVs dysregulated the EC barrier function (**Figures 4J, K**). Furthermore, we examined the vascular permeability by miR-1246 *in vivo* by dextran iv injection. Although there was no significant difference, the vascular permeability was found to be suppressed in tumors in anti-miR-1246 A375SM tumor-bearing mice (**Figures 4L, M**). Collectively, these results suggest that miR-1246 in high metastatic tumor EVs promotes tumor metastasis by disrupting EC barrier function *via* VE-Cadherin downregulation

## 4 Discussion

**Figure 5:**
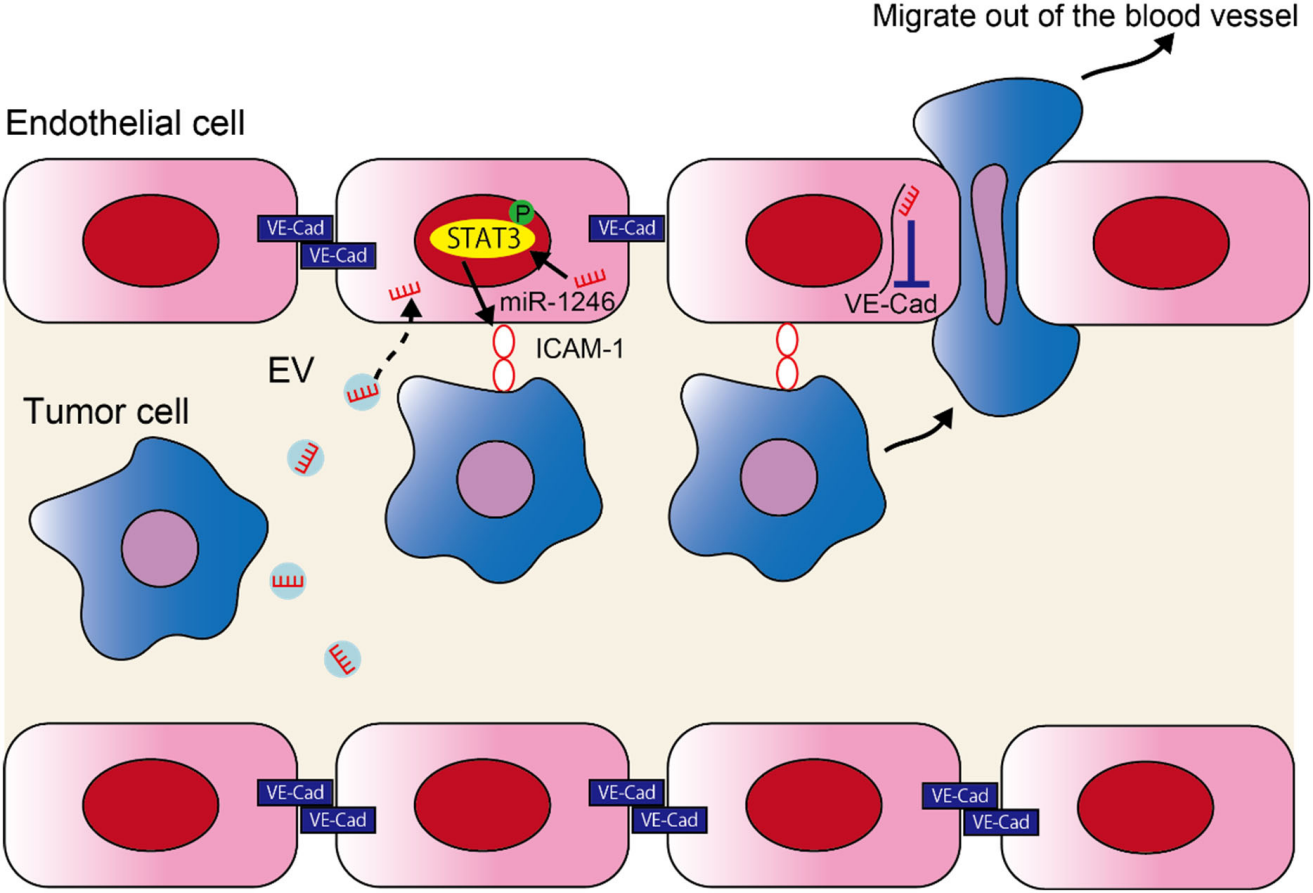
miR-1246 in high metastatic tumor EVs promotes metastasis by promoting the adhesion of tumor cells and destruction of the EC barrier. A schematic diagram of the study. miR-1246 released from high metastatic tumor cells *via* EVs is taken up by ECs and induces ICAM-1 expression *via* STAT3 activation, which promotes tumor cell adhesion to ECs. Furthermore, miR-1246 destroys the EC barrier by suppressing VE-Cadherin expression and promotes the transendothelial migration of tumor cells.

In the current study, we found that miR-1246 in high metastatic tumor EVs increased metastasis by altering the EC phenotype. The following two mechanisms were proposed to explain this (**Figure 5**); 1) miR-1246 is taken up by ECs in high metastatic tumor EVs, thus inducing ICAM-1 expression *via* STAT3 activation in ECs. Consequently, tumor cell adhesion to ECs is promoted. 2) The downregulation of VE-Cadherin expression and destruction of the EC barrier by miR-1246 resulted in the transendothelial migration by tumor cells.

In recent years EVs have attracted attention for their cross-talk function between cells in recent years. Tumor cells utilize EVs secreted in an autocrine manner and induce their migration and invasion (27). Furthermore, tumor cells have been reported to affect stromal cells such as ECs, fibroblasts, and immune cells in the tumor microenvironment by secreting EVs to establish an environment favorable for their development and proliferation. We discovered that ECs take up tumor EVs, and that activation of Akt enhances their angiogenic potential (10). In addition, we have reported that miR-1246 increases IL-6 secretion of ECs in highly metastatic tumor EVs, resulting in induction of drug resistance through the activation of STAT3 and Akt (15). However, it remains unclear the role of miR-1246 in tumor metastasis, especially in vascular ECs. Several studies have examined the role of miRNAs in tumor EVs in metastasis. Zhou et al. reported that miR-105 in breast cancer EVs suppresses the expression of ZO-1 in ECs and destroys the EC barrier, thereby promoting metastasis (14). EVs secreted by hepatoma cell contains miR-103, which promotes metastasis by targeting VE-Cadherin, p120, and ZO-1 in ECs and increasing vascular permeability (28). Tominaga et al. revealed that miR-181c in metastatic breast cancer EVs inhibits actin polymerization of ECs, thereby destroying the blood–brain barrier and promoting brain metastasis in breast cancer (22). However, the precise mechanism by which miR-1246 alters the EC phenotype in tumor-derived EVs is uncertain.

Our study showed that high metastatic tumor EVs, including miR-1246, promoted tumor cell adhesion to the lungs and lung metastasis. Although tumor proliferation was not affected, knockdown of miR-1246 in tumor cells resulted in the decrease of EV-miR-1246 level in the blood and suppression of lung metastasis. These findings suggest that miR-1246 promotes metastasis without affecting tumor growth. Thus, we analyzed the effects of EC phenotype by miR-1246 on blood vessels—a crucial pathway in metastasis. In particular, we analyzed the effect of miR-1246 on tumor cell adhesion between ECs and intracellular adhesion of ECs, which is the EC barrier.

Tumor cells adhere to ECs prior to intravasation at the primary site and extravasation in distant organs. ICAM-1, VCAM-1, and E-selectin act as their adhesion molecules (29). E-selectin is involved in rolling on ECs by leukocytes. ICAM-1 and VCAM-1 are involved in subsequent adhesion with leukocytes, and they play a role in tumor cells–ECs adhesion (30, 31). Induction of ICAM-1, VCAM-1, and E-selectin in ECs has been shown to promote tumor cell adhesion (32–34). Here, we focused on ICAM-1 mainly since it supposed to be upregulated by miR-1246 transfection in ECs. ICAM-1 is expressed on the cell membranes of leukocytes, fibroblasts, and ECs and is induced by the IL-6-STAT3 pathway (24). We previously reported that EV-miR-1246 targets androgen receptor in ECs and induces phosphorylation of STAT3 *via* IL-6 (15). Because STAT3 is one of the transcription factors of ICAM-1, we think that ICAM-1 expression was also induced by the same pathway in this study. Another research group has also been reported that miR-1246 induces IL-6 secretion by targeting PPP2CB and PRKAR1A in mesenchymal stem/stroma cells (35). miR-1246 can induce STAT3 phosphorylation in several cell types. STAT3 activation in ECs is involved in tumor cell adhesion to ECs and metastasis (36). However, the mechanism that miRNAs in tumor EVs promote metastasis by inducing tumor cell adhesion to ECs has not been previously reported.

When tumor cells intravasate or extravasate during metastasis, they migrate between ECs. Intra-EC adhesion consists of adherence and tight junctions (25). VE-Cadherin constituting adherence junction is a calcium-dependent transmembrane protein expressed specifically on ECs.VE-Cadherin has β-catenin and p120-catenin binding domains in the cell and five extracellular cadherin domains. Adjacent ECs adhere to each other by this extracellular domain and control vascular permeability (37). Various cytokines produced by inflammatory activation and VEGF, an angiogenic factor, attenuate EC adhesion (composed of VE-cadherin) and enhance vascular permeability (38). Claudin-5 and ZO-1 constitute the tight junction. Claudin-5 is a transmembrane protein lined with ZO-1 (25). They form the EC barrier together with adherence junction and control vascular permeability. Here, we focused on the VE-Cadherin, whose expression was decreased by miR-1246 transfection.

Zhang et al. found that miR-1246 is involved in tumorigenesis and malignancy of non-small cell lung cancer and inhibiting miR-1246 in tumor cells reduces metastasis (39). Yamada et al. discovered that miR-1246 in colon cancer cell-derived EVs is taken up by ECs, which increased their angiogenic potential through Smad 1/5/8 signal (40). miR-1246 levels in blood EVs have been found to be higher in patients with colorectal cancer, breast cancer, and pancreatic cancer than in healthy volunteers, suggesting that miR-1246 can be a biomarker (41–43). However, there was no report that examined how miR-1246 alters the EC phenotype in connection to metastasis. Furthermore, no miRNAs have been reported to affect the two critical pathways of metastasis: tumor cell adhesion to ECs and disruption of the EC barrier.

However, it is unknown if EVs are taken up by lung ECs in our study. Tumor EVs reflect the organ specificity of the EV secreting tumor cells. As the mechanisms, it is reported that integrin expression patterns in the tumor EVs are involved in the organ tropism (44). In addition, brain metastatic cancer cell-derived EVs were incorporated into ECs but not pericytes or astrocytes (22). Tumor cells that we used in our study metastasized to the lung, and their EVs were likely taken up by lung ECs, which will be shown in future studies. Nishida-Aoki et al. reported that the glycosylation profile of EVs affects the uptake by ECs, and the profile differences among the EVs we used may be related to metastatic potential (45). Furthermore, the effect of miR-1246 on tumor cells themselves or other stromal cells such as immune cells and fibroblasts in the tumor microenvironment was not analyzed; therefore, it would be included in further research. In addition, EVs contain many proteins, mRNAs, and miRNAs; therefore, we cannot rule out the possibility of molecules besides miR-1246 as the cause of EC character change in this study. However, our findings that miR-1246 knockdown tumor cell EVs suggested that miR-1246 inhibition in ECs is one of the mechanisms of decreasing metastasis.

Our study suggests that metastasis can be prevented by targeting tumor cell-derived EVs containing miR-1246. Since our previous report—where EV-miR-1246 levels in the blood of patients with melanoma are high (15)—miR-1246 is expected to be a biomarker for early diagnosis of cancer. miR-1246 could be used for the diagnosis and as a target for tumor metastasis.

## Data availability statement

The original contributions presented in the study are included in the article/**Supplementary Materials**, further inquiries can be directed to the corresponding author/s

## Ethics statement

The animal study was reviewed and approved by Experimental Animal Care of Hokkaido University.

## Author contributions

MM performed and analyzed the experiments and wrote the manuscript. TT, MA, and RT contributed to the completion of various experiments. HK, YH, YY, TO, DA, and YK provided some important suggestions. NM and KH designed the study and wrote the manuscript. All authors read and approved the final manuscript.

## Supplementary material

Supplementary Figure 1 EV characterization. **(A)** A375SM-EVs were observed by a transmission electron microscope. Scale bar: 100 nm. **(B)** The particle size distribution of A375SM-EVs was measured by dynamic light scattering. **(C)** The levels of HSP70 and cytochrome C in A375SM-EVs were determined by western blotting.

Supplementary Figure 2 miR-1246 levels in miR-1246-knockdown tumor cells. miR-1246 levels in anti-miR-1246 A375SM cells **(A)** and EVs **(B)** were examined by qRT-PCR. Data are presented as mean ± SD; n = 3 real-time RT-PCR runs (*P < 0.0001 vs. control-miR-1246 A375SM, two-sided Student’s t-test).

Supplementary Figure 3 ICAM-1 induction by tumor-EV under the STAT3 inhibitor. ICAM-1 mRNA levels in iHMVECs treated with A375SM-EVs with or without the STAT3 inhibitor (S3I-201) were examined by qRT-PCR (*P =0.0009 (Control vs. A375SM-EV), 0.0002 (A375SM-EV vs. S3I-201(10μM) + A375SM-EV), 0.0048 (S3I-201(10μM) + A375SM-EV vs. S3I-201(50μM) + A375SM-EV), one-way ANOVA, followed by a Tukey–Kramer multiple comparison tests). PBS was used as the control. Data are presented as mean ± SD; n = 4 real-time RT-PCR runs.

Supplementary Figure 4 miR-1246 in EVs promotes the adhesion of high metastatic tumor cell to ECs, related to . **(A)** Representative fluorescent images of adherent tdTomato-luc2-expressing A375SM cells to iHMVEC monolayer treated with A375SM-EVs. The lower panel shows an enlarged image of the regions marked with yellow rectangles. PBS was used as the control. **(B)** The number of tdTomato-luc2-expressing A375SM cells was counted. Data are presented as mean ± SD; n = 5 fields. Scale bar: 100 µm (*P < 0.0001 vs. control, two-sided Student’s t-test). **(C)** Representative fluorescent images of adherent tdTomato-luc2-expressing A375SM cells to miR-1246 transfected iHMVEC monolayer. The lower panel shows an enlarged image of the regions marked with yellow rectangles. microRNA Mimic Negative Control was used as the control. **(D)** The number of tdTomato-luc2-expressing A375SM cells was counted. Data are presented as mean ± SD; n = 5 fields. Scale bar: 100 µm (*P < 0.0001 vs. control, two-sided Student’s t-test). **(E)** Representative fluorescent images of adherent tdTomato-luc2-expressing A375SM cells to iHMVEC monolayer treated with Anti-miR-1246 A375SM-EVs. The lower panel shows an enlarged image of the regions marked with yellow rectangles. **(F)** The number of tdTomato-luc2-expressing A375SM cells was counted. Data are presented as mean ± SD; n = 5 fields. Scale bar: 100 µm (*P < 0.0001 vs. control-miR A375SM-EVs, two-sided Student’s t-test).
