# Supplementary figures and images for: miR-1246 in tumor extracellular vesicles promotes metastasis via increased tumor cell adhesion and endothelial cell barrier destruction
Source: Front Oncol. 2023 Apr 12;13:973871. doi: 10.3389/fonc.2023.973871 (PMC10130374; doi:10.3389/fonc.2023.973871)

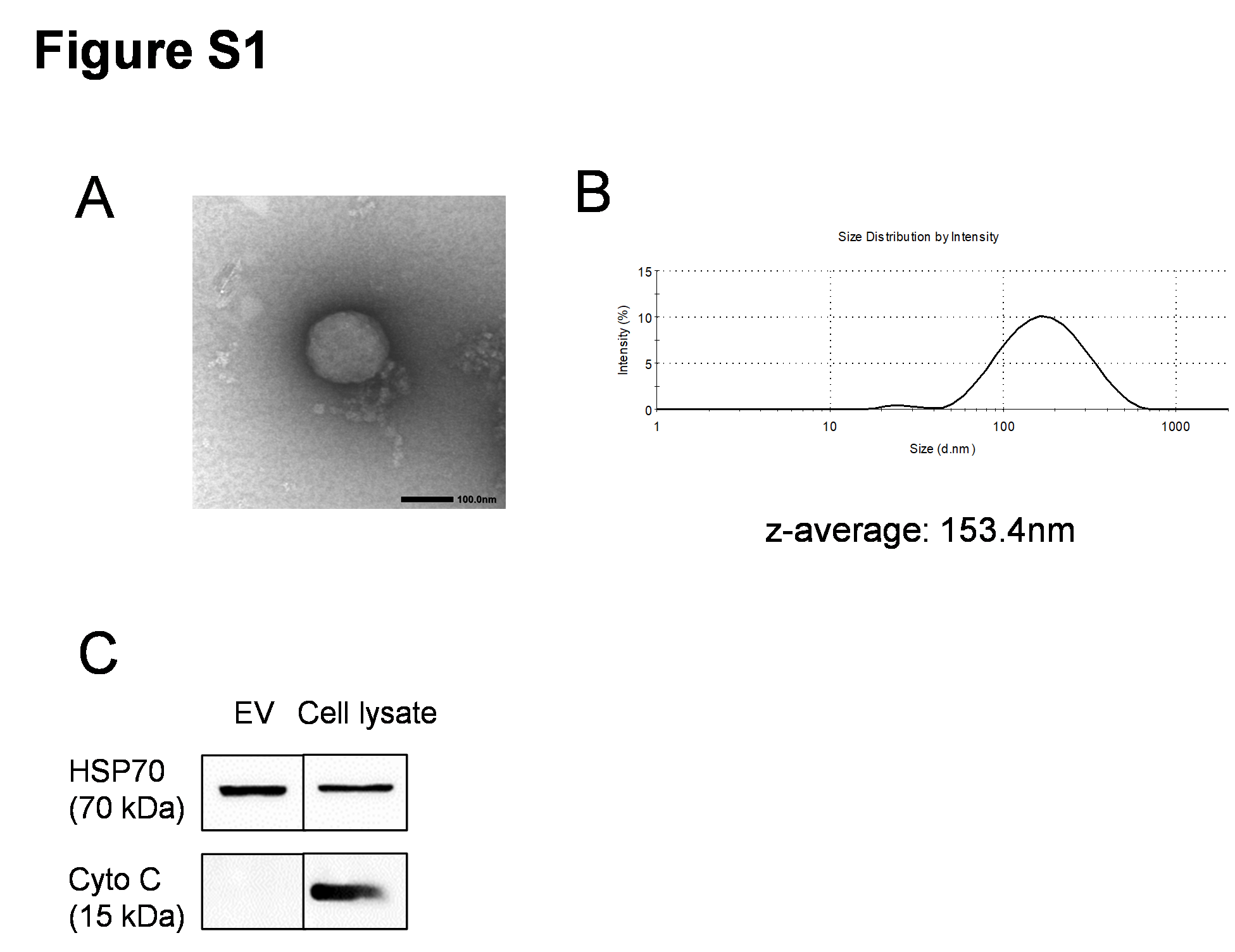

Supplement: Supplementary Figure 1 — EV characterization. (A) A375SM-EVs were observed by a transmission electron microscope. Scale bar: 100 nm. (B) The particle size distribution of A375SM-EVs was measured by dynamic light scattering. (C) The levels of HSP70 and cytochrome C in A375SM-EVs were determined by western blotting. [file Image_1.tif]

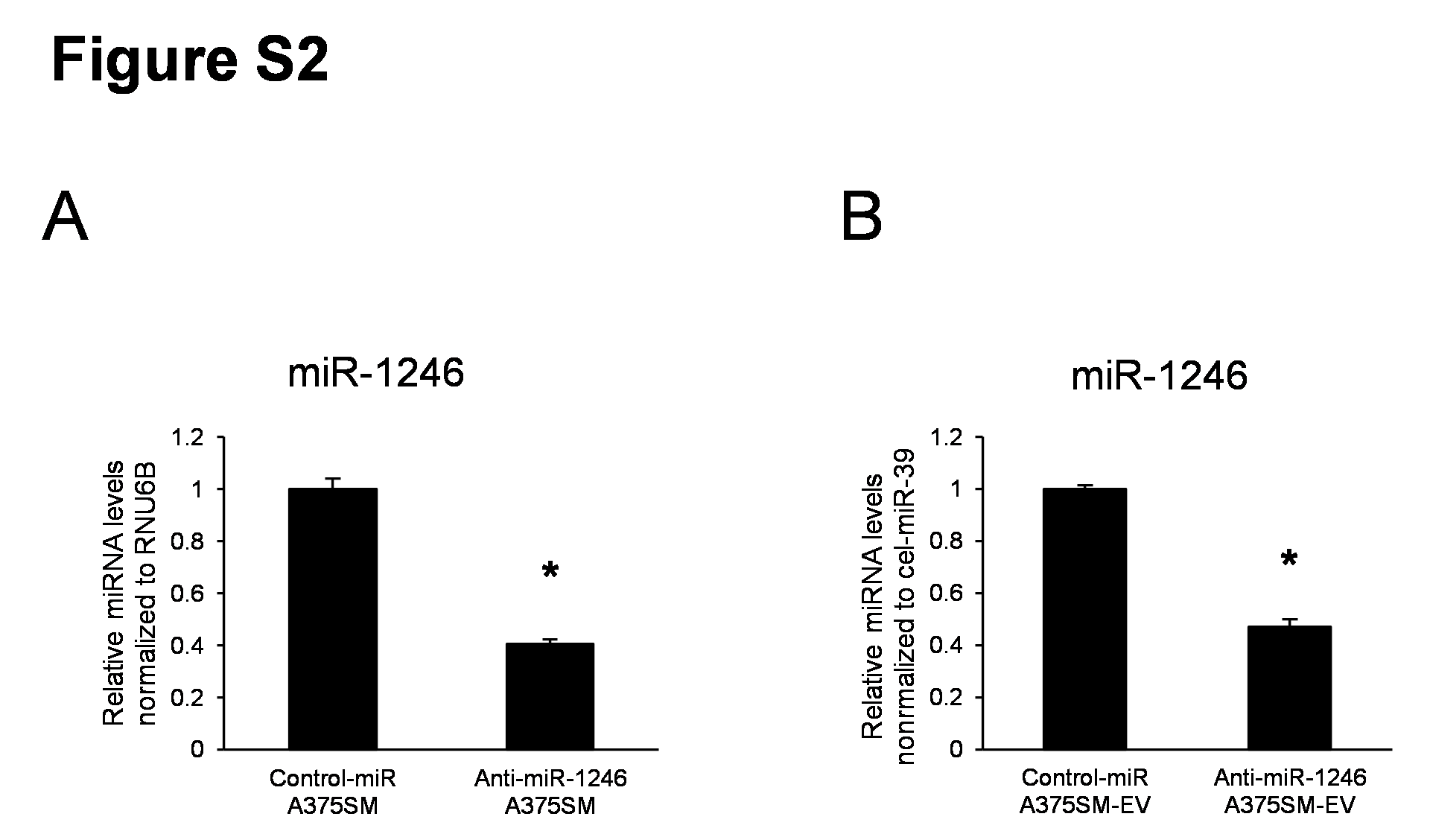

Supplement: Supplementary Figure 2 — miR-1246 levels in miR-1246-knockdown tumor cells. miR-1246 levels in anti-miR-1246 A375SM cells (A) and EVs (B) were examined by qRT-PCR. Data are presented as mean ± SD; n = 3 real-time RT-PCR runs (*P < 0.0001 vs. control-miR-1246 A375SM, two-sided Student’s t-test). [file Image_2.tif]

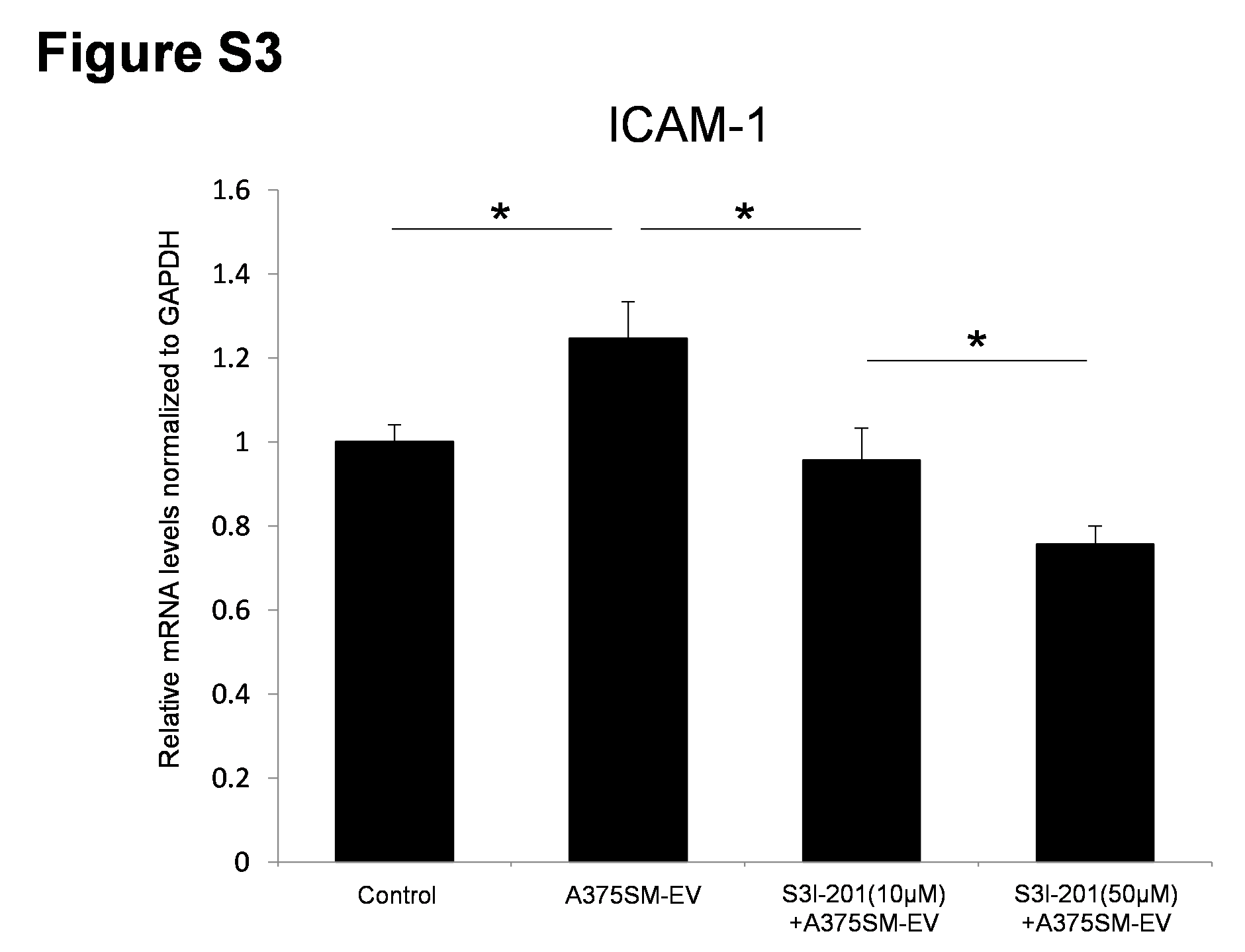

Supplement: Supplementary Figure 3 — ICAM-1 induction by tumor-EV under the STAT3 inhibitor. ICAM-1 mRNA levels in iHMVECs treated with A375SM-EVs with or without the STAT3 inhibitor (S3I-201) were examined by qRT-PCR (*P =0.0009 (Control vs. A375SM-EV), 0.0002 (A375SM-EV vs. S3I-201(10μM) + A375SM-EV), 0.0048 (S3I-201(10μM) + A375SM-EV vs. S3I-201(50μM) + A375SM-EV), one-way ANOVA, followed by a Tukey–Kramer multiple comparison tests). PBS was used as the control. Data are presented as mean ± SD; n = 4 real-time RT-PCR runs. [file Image_3.tif]

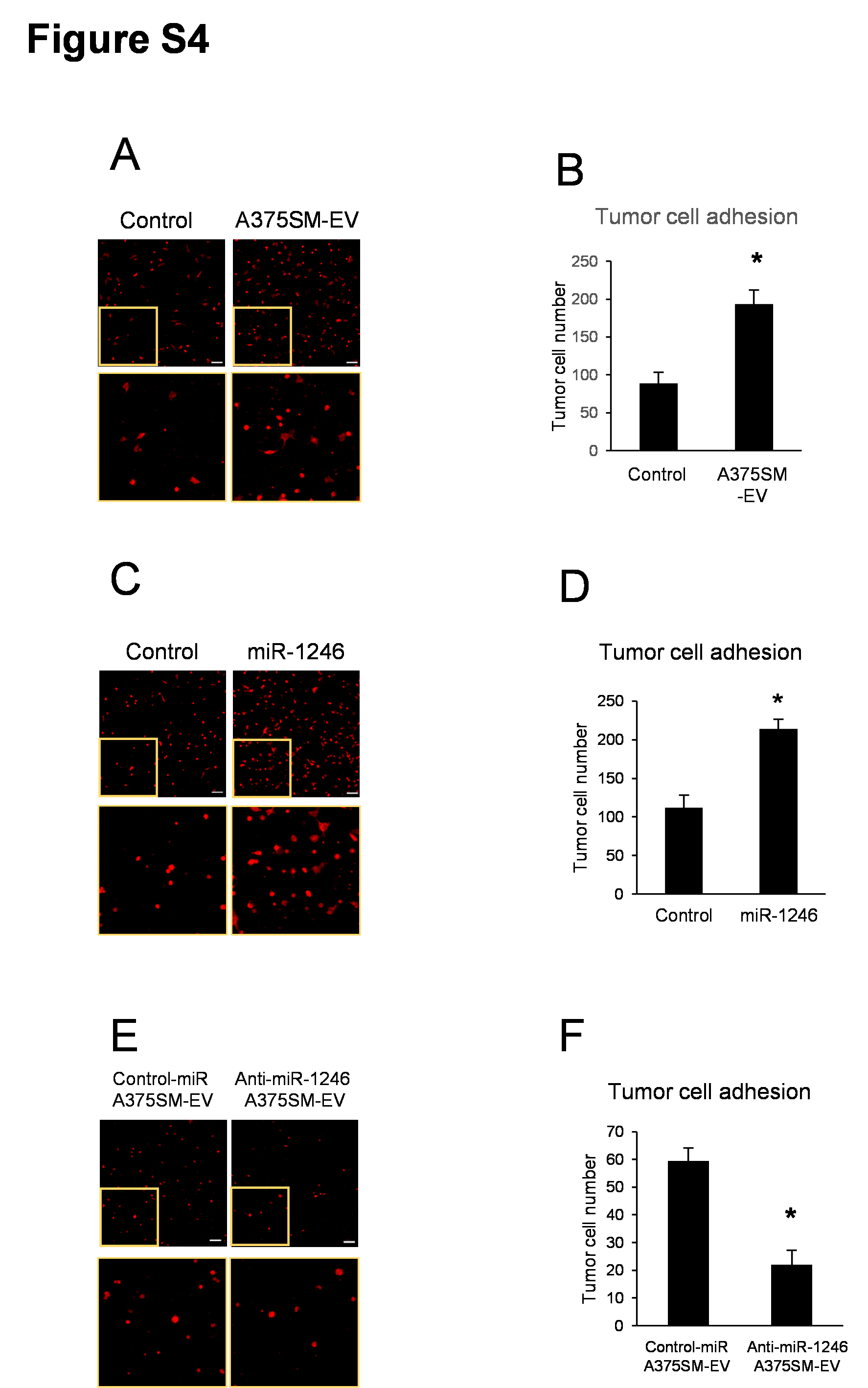

Supplement: Supplementary Figure 4 — miR-1246 in EVs promotes the adhesion of high metastatic tumor cell to ECs, related to . (A) Representative fluorescent images of adherent tdTomato-luc2-expressing A375SM cells to iHMVEC monolayer treated with A375SM-EVs. The lower panel shows an enlarged image of the regions marked with yellow rectangles. PBS was used as the control. (B) The number of tdTomato-luc2-expressing A375SM cells was counted. Data are presented as mean ± SD; n = 5 fields. Scale bar: 100 µm (*P < 0.0001 vs. control, two-sided Student’s t-test). (C) Representative fluorescent images of adherent tdTomato-luc2-expressing A375SM cells to miR-1246 transfected iHMVEC monolayer. The lower panel shows an enlarged image of the regions marked with yellow rectangles. microRNA Mimic Negative Control was used as the control. (D) The number of tdTomato-luc2-expressing A375SM cells was counted. Data are presented as mean ± SD; n = 5 fields. Scale bar: 100 µm (*P < 0.0001 vs. control, two-sided Student’s t-test). (E) Representative fluorescent images of adherent tdTomato-luc2-expressing A375SM cells to iHMVEC monolayer treated with Anti-miR-1246 A375SM-EVs. The lower panel shows an enlarged image of the regions marked with yellow rectangles. (F) The number of tdTomato-luc2-expressing A375SM cells was counted. Data are presented as mean ± SD; n = 5 fields. Scale bar: 100 µm (*P < 0.0001 vs. control-miR A375SM-EVs, two-sided Student’s t-test). [file Image_4.tif]
